# Supplementary material for: Invadosome Formation by Lung Fibroblasts in Idiopathic Pulmonary Fibrosis
Source: Int J Mol Sci. 2022 Dec 28;24(1):499. doi: 10.3390/ijms24010499 (PMC9820272; doi:10.3390/ijms24010499)
Supplement: Supplementary file 1 [file ijms-24-00499-s001.zip › ijms-2010746-supplementary.docx]

**Invadosome Formation by Lung Fibroblasts in Idiopathic Pulmonary Fibrosis**

Megane Lebel.^1^, Dominic O. Cliche.^1^, Martine Charbonneau^.2^, Damien Adam^3^, Emmanuelle Brochiero^3,4^, Claire M. Dubois^2^ and André M. Cantin^1^

^1^Respiratory Division, Department of Medicine, ^2^Department of Immunology and Cell Biology, Université de Sherbrooke, Sherbrooke, Québec, Canada, ^3^Centre de recherche du Centre hospitalier de l’Université de Montréal (CRCHUM) and ^4^Department of Medicine, Université de Montréal, Montréal, Québec, Canada

**DATA SUPPLEMENT**

**Supplementary material & methods**

**Reagents:** Bleomycin was from Hospira Healthcare Corporation. Ketamine and Xylazine were from CDMV inc. Liberase TM of research grade was from Roche. Nintedanib was from Selleckchem (Burlington, Canada). Pirfenidone was purchased from Toronto Research Chemicals (North York, Canada). PP2 (AG1879) was from Aobious and human PDGF-BB from Peprotech. Gelatin pig skin Oregon Green 488 conjugate, goat anti-rabbit (A11008) and anti-mouse (A11001) 488 conjugate, DAPI and phalloidin Texas Red were from Invitrogen. The rabbit anti-cortactin antibody (ab11065-5) was obtained from Abcam. Mouse anti-TE-7 (CBL271) and mouse anti-TKS5 (MABT336) antibodies was purchased from EMD Millipore. Rabbit anti-Akt (9272S), rabbit anti-Src (2108S), rabbit anti-phospho-Src (2101S), rabbit anti-phospho-Akt (9271S) and rabbit anti-phospho-cortactin (4569) antibodies were purchased from Cell Signaling. Rabbit anti-TKS5 (NBP1-90454) was from Novus Biologicals. Rabbit anti-fibronectin (F3648) was obtained from Sigma and rabbit anti-E-cadherin (13-1700) from Calbiochem. Diaminobenzidine (DAB) was from DAKO. Streptavidin-HRP and biotin-SP goat anti-rabbit (111-065-003) were obtained from Jackson Immunoresearch Laboratories.

**Processing of a public gene expression study**: Gene expression matrix from GSE32537 dataset (<https://www.ncbi.nlm.nih.gov/bioproject/PRJNA154433>) and GSE169500 dataset (<https://www.ncbi.nlm.nih.gov/bioproject/?term=GSE169500>) were downloaded from Gene Expression Omnibus. A list of invadosome-associated genes was established based on previous works [1-4]. Normalized expression data were transformed into a Z score and displayed on heatmaps. For multiple testing, p-values were determined with a Mann-Whitney test following with a Benjamini-Hochberg false discovery rate adjustment. Adjusted p-values less than 0.05 were considered significant. Differential gene analysis was assessed with the Control (n=50) and IPF (n=119) subsets of the GSE32537 cohort and 17 selected genes of interest are presented. Six other genes associated with fibrosis were also selected. Gene expression correlations were assessed using the IPF subset (n=119) and correlation strength was measured with a two-tailed Spearman’s test. Differential gene analysis was also assessed with Control alveolar septae (n=10), IPF alveolar septae (n=10) and IPF fiboblastic foci (n=10) subsets of the GSE169500 cohort and 10 selected gene of interest are presented.

**Lentiviral production and transduction:** Performed as described previously [5]. Lentivirus with shRNA targeting TKS5 (MISSION shRNA #TRCN0000425725, Sigma) or scramble sequence were generated by transfecting HEK 293T cells with the Virapower lentiviral expression system. IPF fibroblasts were transduced with TKS5 or scramble shRNA using 1mL of lentiviral stock, 2mL of optiMEM and 1µL of Polybrene (3µg/mL). A positive selection was performed using puromycin (5µg/mL).

**Flow cytometry:** Following cell dissociation, 5x10^5^ cells were fixed with paraformaldehyde (PFA, 1%) and permeabilized with saponin (0.05%). The primary antibodies TE-7, fibronectin and E-cadherin were incubated 1h and secondary antibodies 30 minutes one ice. Cells were filtered using a 70 µm mesh and analysed using a Becton Dickinson Cytoflex 15 Analyser within one hour. Results are expressed as the percentage of positive cells or the GeoMean fluorescence intensity (MFI).

**Murine lung fibrosis model:** All experiments were performed in accordance with the animal use protocol approved by the Ethics Committee on Animal Research of the Université de Sherbrooke (CFPA-FMSS) and the guidelines of the Canadian Council on Animal Care. Eight to twelve-week- old C57BL/6 male mice were obtained from Charles River Laboratories. Mice were anesthetized with a combination of ketamine and xylazine prior to a single intratracheal injection of 1 U/kg of bleomycin. Lungs were collected for fibroblast isolation at 28 days post-bleomycin challenge. Lungs from healthy mice were also collected and used as a control.

**mRNA expression analysis:** Fibroblasts were grown to 80% confluency and then starved with serum-free DMEM for 16 hours before RNA extraction (RiboZol™ reagent, VWR Life Sciences). RNA was reverse transcribed (iScript™ supermix, Bio-Rad Laboratories) using 0.5µg per reaction. TKS5, COL1A1, CTGF and RPL13 transcripts **(Table 2)** were amplified by qPCR using SYBR Green qPCR master mix (BiMake) and Rotor-Gene 3000 (Corbett Research, Kirkland, Canada). RPL13 was employed as the housekeeping control to calculate 2-∆∆Ct. Results were presented as a fold increase referring to the mean of control condition.

**Immunoblotting:** Fibroblasts were grown to 80% confluency and then starved with serum-free DMEM for 16 hours. Proteins were harvested using lysis buffer (Tris-HCl 50 mM, NaCl 150 mM, Na-deoxycholate 0.1%, EDTA 4 mM, NP-40 1%) containing phosphatase and protease inhibitors. Cell lysates (15-20 µg of total protein) were loaded on 10% SDS-PAGE gels. Proteins were detected using ChemiDoc MP Imager (Bio-Rad Laboratories). Band intensity was quantified using Image Lab software (Bio-Rad Laboratories).

**Figures and figure legends**


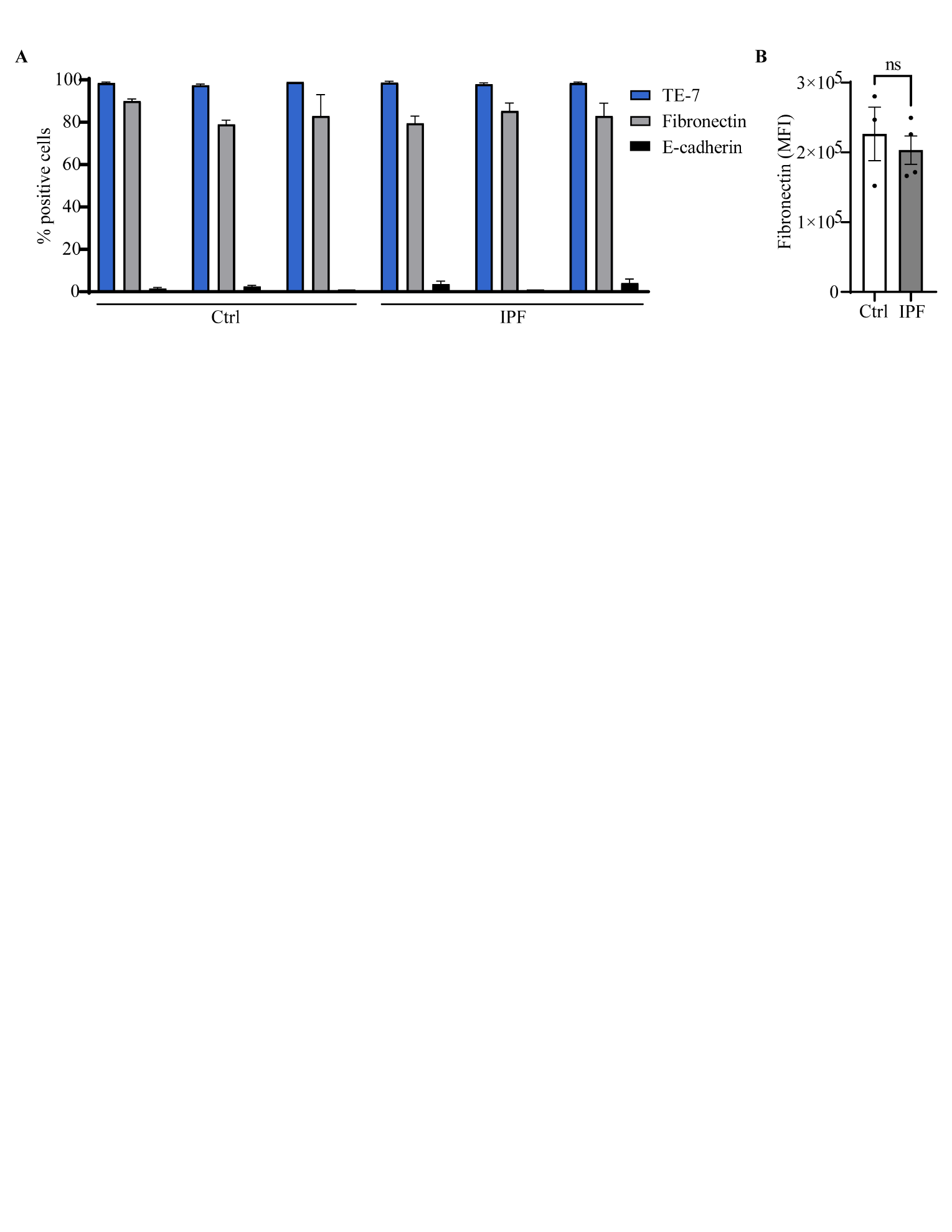
**Figure S1. Authentication of the fibroblast cultures isolated from healthy donors and IPF patients**. **A)** Three control and three IPF cell cultures were analysed by flow cytometry with the fibroblastic markers TE-7 and fibronectin, and the epithelial maker E-cadherin (n=2-4). **B)** Fibronectin expression (MFI) in control (ctrl) and IPF cell cultures. ns= non significant.


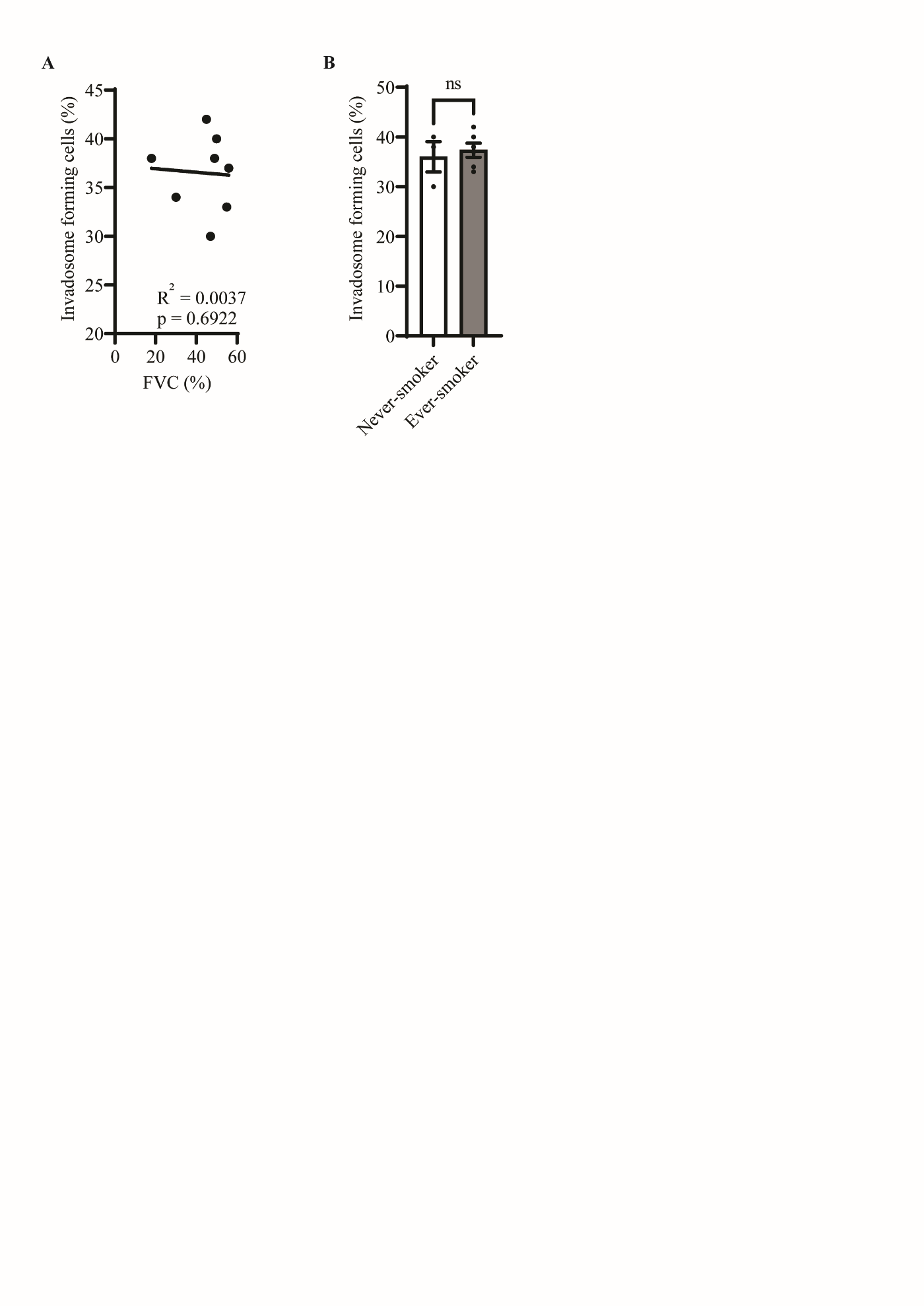


**Figure S2. The percentage of invadosome forming cells is not associated with the FVC or smoking status. A)** Forced vital capacity (FVC) *versus* the percentage of invadosome formation by lung base-derived fibroblasts (n=8). **B)** Invadosome formation according to the smoking status of IPF patients. Never-smoker (n=3) and ever-smoker (n=6).


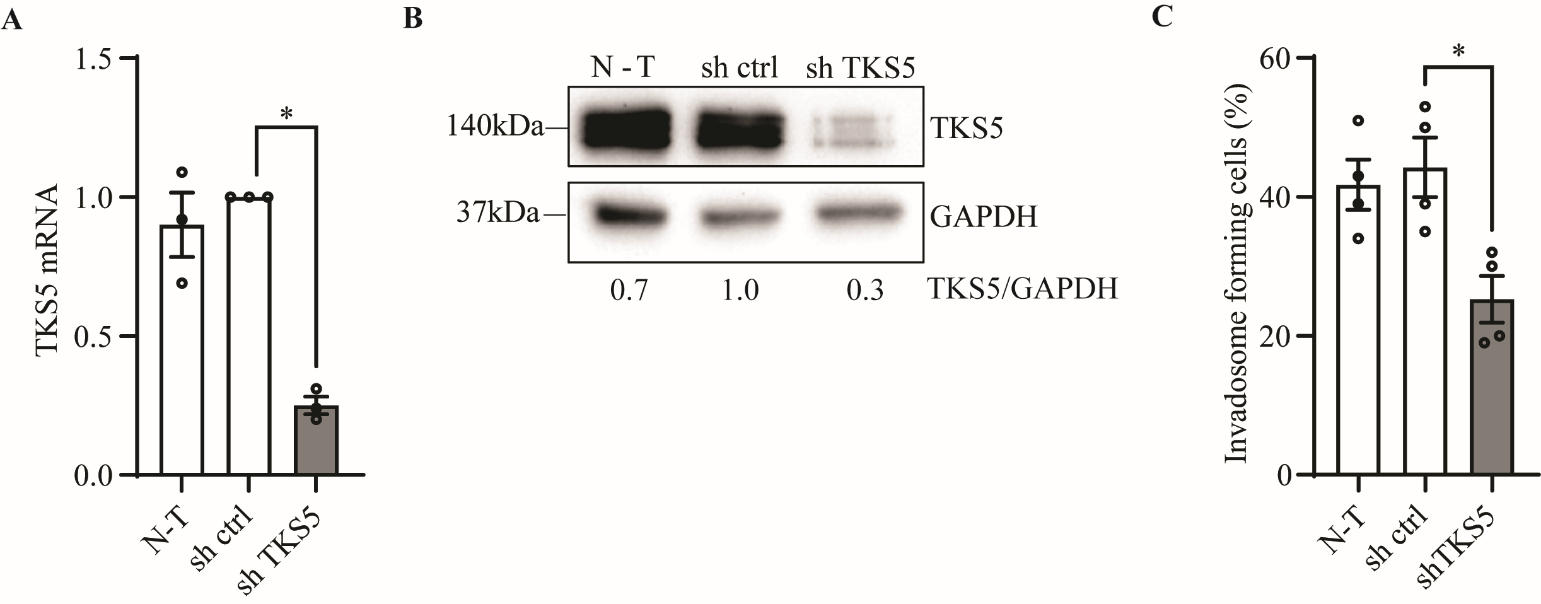


Figure S3: Inhibition of TKS5 impaired the formation of invadosomes by IPF fibroblasts. IPF fibroblasts were non-transduced (N-T) or transduced with a scramble (ctrl) or TKS5 targeting shRNA. A) mRNA and B) protein levels of TKS5 relative to sh ctrl (n=3). C) Percentage of invadosome-forming cells (n=4). *p<0.05.


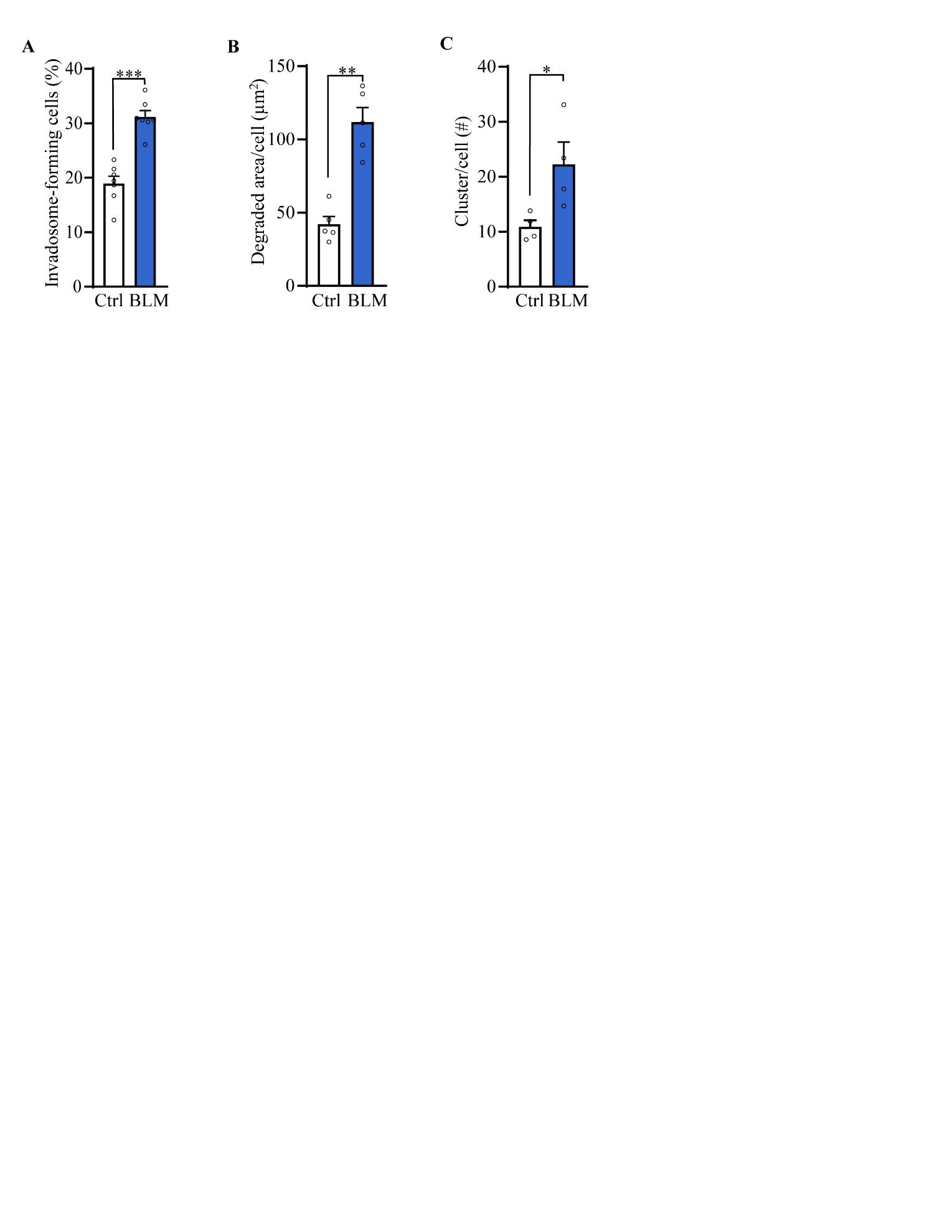
**Figure S4.** **Invadosome formation was also assessed in mouse fibroblasts isolated from the bleomycin (BLM)-induced fibrosis model**. **A)** Invadosome formation frequency (n=7), **B)** degradation capacity (n=5) and **C)** number of invadosomal structures (n=5) were evaluated.


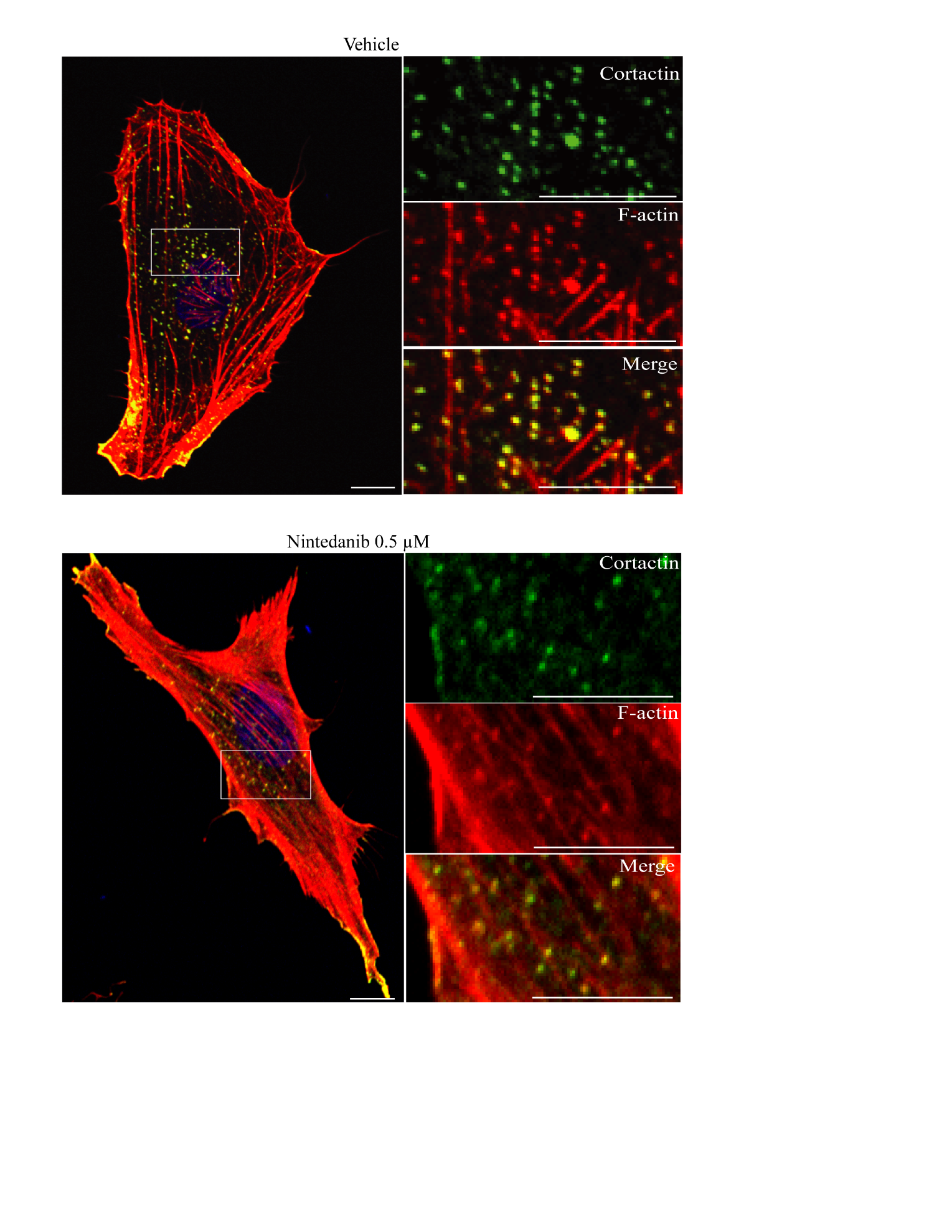
**Figure S5.** **Nintedanib decreases the assembly of cortactin and f-actin in IPF fibroblasts.** Confocal microscopy captures of fibroblasts incubated in the presence or absence of nintedanib stained for cortactin (green), f-actin (red) and nucleus (blue). An invadosome-rich region is zoomed in and presented on the right. Scale bar = 10µm.


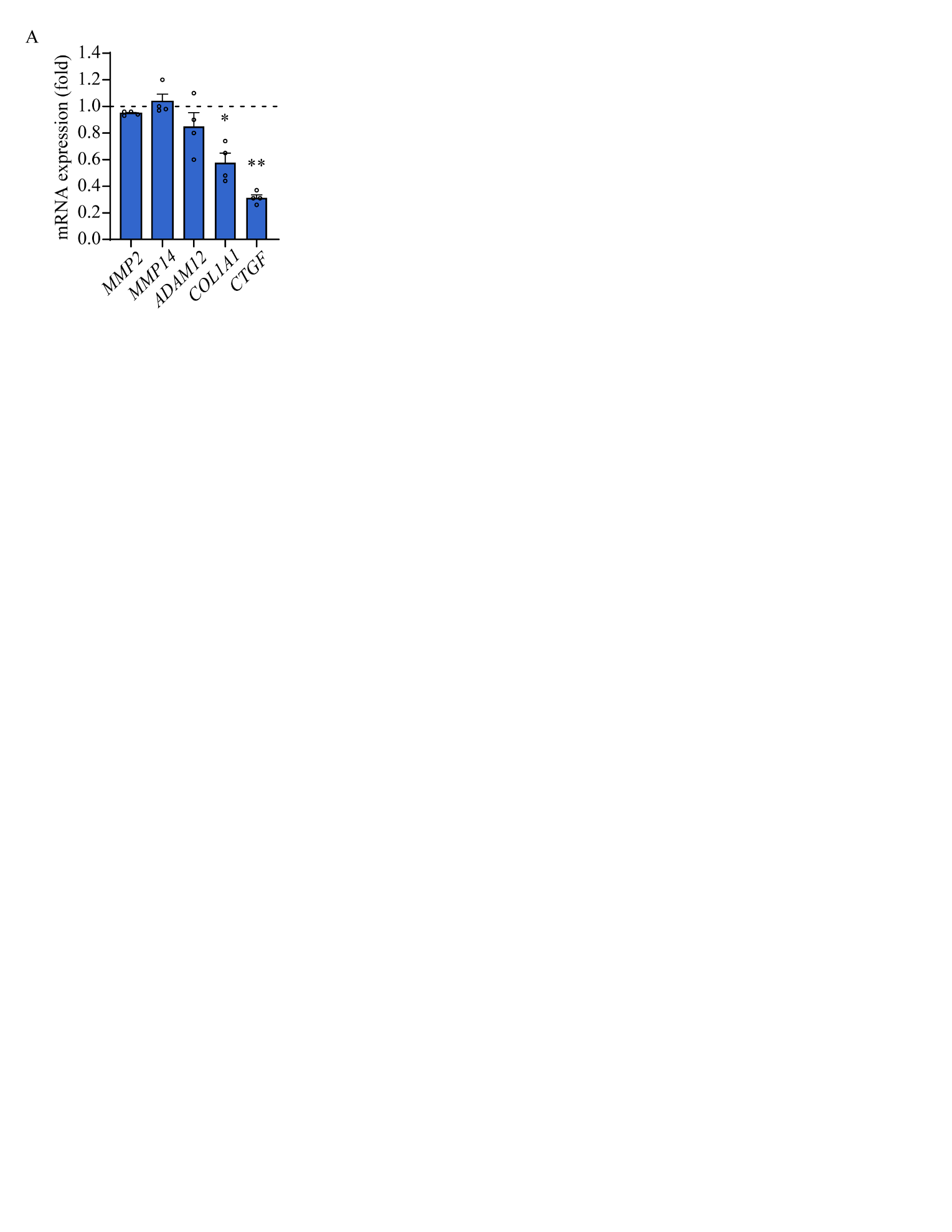
 **Figure S6. Nintedanib has no effect on the level of *MMP2*, *MMP14* and *ADAM12* gene expression**. IPF fibroblasts were cultured for 24h with nintedanib 0.5µM and mRNA levels of matrix metalloproteiase 2 and 14 (*MMP2, MMP14*), disintegrin and metalloproteinase domain-containing protein 12 (*ADAM12*), type I collagen (*COL1A1*) and connective tissue growth factor (*CTGF*) were measured and expressed in folds relative to untreated cells (n=4).


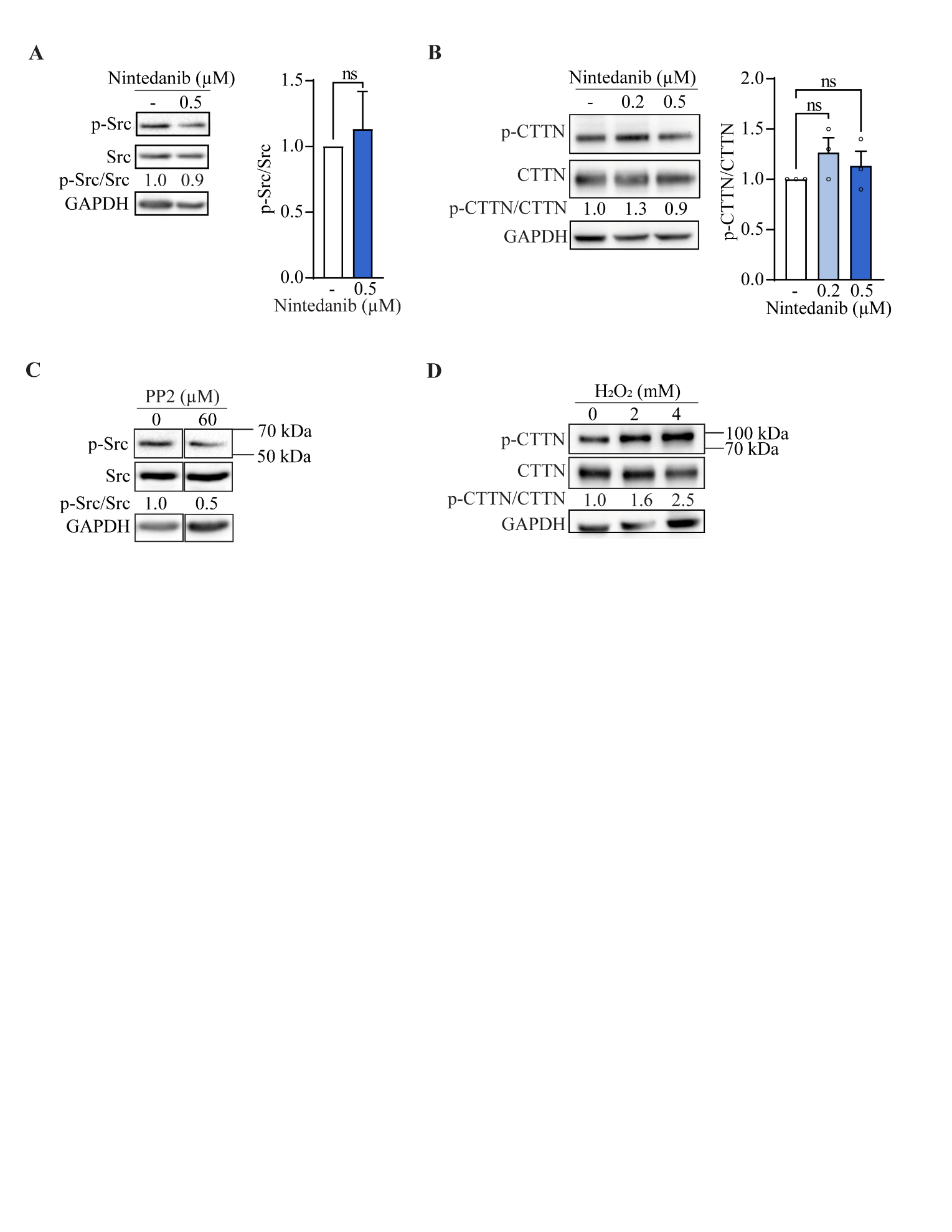
**Figure S7.** **Nintedanib has no effect on the level of Src and cortactin (CTTN) phosphorylation**. **A)** Immunoblot of p-Src (Y416) and **B)** p-CTTN (Y421) with lysates of IPF lung fibroblasts cultured with nintedanib 0.2µM and/or 0.5µM (n=3). **C)** validation of pY416-Src (60kDa) antibody using IPF fibroblasts treated with the Src inhibitor PP2. **D)** Validation of pY421-CTTN (80-85kDa) antibody using IPF fibroblasts treated with H_2_O_2_.

**Reference**

1. Murphy, D.A.; Courtneidge, S.A. The 'ins' and 'outs' of podosomes and invadopodia: characteristics, formation and function. *Nature Reviews Molecular Cell Biology* **2011**, *12*, 413-426, doi:10.1038/nrm3141.

2. Ninio, L.; Nissani, A.; Meirson, T.; Domovitz, T.; Genna, A.; Twafra, S.; Srikanth, K.D.; Dabour, R.; Avraham, E.; Davidovich, A.; et al. Hepatitis C Virus Enhances the Invasiveness of Hepatocellular Carcinoma via EGFR-Mediated Invadopodia Formation and Activation. *Cells* **2019**, *8*, doi:10.3390/cells8111395.

3. Cambi, A.; Chavrier, P. Tissue remodeling by invadosomes. *Fac Rev* **2021**, *10*, 39, doi:10.12703/r/10-39.

4. Saini, P.; Courtneidge, S.A. Tks adaptor proteins at a glance. *J Cell Sci* **2018**, *131*, doi:10.1242/jcs.203661.

5. Brochu-Gaudreau, K.; Charbonneau, M.; Harper, K.; Dubois, C.M. Hypoxia Selectively Increases a SMAD3 Signaling Axis to Promote Cancer Cell Invasion. *Cancers (Basel)* **2022**, *14*, doi:10.3390/cancers14112751.
